# Folic acid-modified nanocrystalline cellulose for enhanced delivery and anti-cancer effects of crocin

**DOI:** 10.1038/s41598-024-64758-2

**Published:** 2024-06-17

**Authors:** Mozhgan Soltani, Amin Farhadi, Sarah Rajabi, Masoud Homayouni‐Tabrizi, Fatimah Sameer Hussein, Navid Mohammadian

**Affiliations:** 1 Department of Biology, Mashhad Branch, Islamic Azad University, Mashhad, Iran; 2 Department of Biology, Science and Research Branch, Islamic Azad University, Tehran, Iran; 3 Department of Tissue Engineering, School of Advanced Technologies in Medicine, Royan Institute, Tehran, Iran; 4 Department of Cell Engineering, Cell Science Research Center, Royan Institute for Stem Cell Biology and Technology, ACECR, Tehran, Iran; 5 Department of Chemistry, Mashhad Branch, Islamic Azad University, Mashhad, Iran

**Keywords:** Crocin, Saffron, Anti-cancer, Nanocrystal cellulose, Folic acid, Apoptosis, Biochemistry, Biotechnology, Cancer, Cell biology, Drug discovery, Medical research

## Abstract

Crocin is a carotenoid compound in saffron with anti-cancer properties. However, its therapeutic application is limited by its low absorption, bioavailability, and stability, which can be overcome through nanocarrier delivery systems. This study used surface-modified Nano-crystalline cellulose (NCC) to deliver crocin to cancer cells. NCC modified with CTAB were loaded with crocin and then conjugated with folic acid (NCF-CR-NPs). The synthesized nanoparticles (NPs) were characterized using FTIR, XRD, DLS, and FESEM. The crystallinity index of NCC was 66.64%, higher than microcrystalline cellulose (61.4%). The crocin loading and encapsulation efficiency in NCF-CR-NPs were evaluated. Toxicity testing by MTT assay showed that NCF-CR-NPs had higher toxicity against various cancer cell lines, including colon cancer HT-29 cells (IC50 ~ 11.6 μg/ml), compared to free crocin. Fluorescent staining, flow cytometry, and molecular analysis confirmed that NCF-CR-NPs induced apoptosis in HT-29 cells by increasing p53 and caspase 8 expression. The antioxidant capacity of NCF-CR-NPs was also evaluated using ABTS and DPPH radical scavenging assays. NCF-CR-NPs exhibited high free radical scavenging ability, with an IC50 of ~ 46.5 μg/ml for ABTS. In conclusion, this study demonstrates the potential of NCF-CR-NPs to deliver crocin to cancer cells effectively. The NPs exhibited enhanced anti-cancer and antioxidant activities compared to free crocin, making them a promising nanocarrier system for crocin-based cancer therapy.

## Introduction

Traditional cancer treatments, though clinically effective, often suffer from significant side effects and the risk of disease recurrence^1^. To address these limitations, new techniques like immunotherapy, targeted therapy, and hormone therapy have been developed^2^. In this regard, the development of drug-delivery nanosystems has emerged as a promising approach to enhancing the therapeutic efficacy and safety of cancer treatments^3^. Nanoparticle (NP)-based delivery systems offer advantages such as improved bioavailability, biocompatibility, and the ability to encapsulate and directly target therapeutic agents to the tumor site^4^. Moreover, these nanocarriers can enhance the stability of the encapsulated drugs during blood circulation^5^, improving their pharmacokinetic profile and reducing off-target toxicity^6,7^. Polymers, particularly cellulose, are widely used in the pharmaceutical industry due to their stability and the ability to modify their surface properties for improved drug delivery^8^. Nanocellulose, with its unique characteristics, excellent biocompatibility, biodegradability, and low toxicity, has emerged as an effective drug delivery system, allowing for the controlled release of therapeutic agents^9^.

Nano-crystalline cellulose (NCC), cellulose nanocrystals (CNCs), or nanocellulose, is a nanomaterial derived from natural cellulose, with a range of advantageous biological properties that make it suitable for various biomedical applications. Cellulose is the most abundant organic polymer on Earth, found in plants, wood, and certain algae and bacteria cell walls. It is composed of linear glucose polymers linked by β-1,4-glycosidic bonds, forming parallel sheets stabilized by hydrogen bonds. NCC is produced by extracting highly ordered, crystalline regions through various mechanical, chemical, or enzymatic treatments^10^. It exhibits excellent biocompatibility and biodegradability, a high surface area-to-volume ratio for efficient delivery of therapeutic agents, versatile surface chemistry enabling targeted drug delivery, remarkable mechanical strength for reinforcing composite biomaterials, intrinsic antimicrobial activity, and optical transparency for potential use in ophthalmic and wound dressing applications. These unique characteristics of NCC establish it as a highly versatile material with great potential in fields such as drug delivery, tissue engineering, wound healing, and antimicrobial coatings. Nevertheless, NCC is not a suitable carrier for hydrophobic and non-ionized compounds due to their hydrophilic nature, so modifying the surface of these systems is an effective step in loading hydrophobic drugs. The use of cetyltrimethylammonium bromide (CTAB) to modify the surface of NCCs can help increase the hydrophobic properties of NPs, and adjust the loading and release of drugs on these drug delivery systems^11^. CTAB is a surfactant with a long hydrophobic tail and a hydrophilic head commonly used to stabilize NPs in solution. CTAB can increase drug binding to these nanostructures and improve their physicochemical and biological properties by modifying the hydroxyl or sulfate groups on NCCs^12^. In addition, the numerous hydroxyl groups on the surface of NCCs can be chemically modified for ligand targeting and conjugation with imaging probes and drugs^13^.

Receptor-mediated cell targeting is one of the effective strategies for early cancer diagnosis and targeted therapy^14^. One of the most well-known receptors of cancer cells is folate receptors (FRs), which are responsible for the cellular absorption of folic acid (FA) (vitamin B9). High expression of these receptors has been confirmed in the plasma membrane of some cancer cells (breast, ovarian, lung, kidney, brain, and endometrial cancer), while normal tissues rarely express FRs^15,16^. The use of FA ligand on the surface of carriers due to the high tendency of this ligand to bind to its receptor on the surface of cancer cells can cause active transfer and internalization of drug delivery systems specifically to cancer cells. This type of internalization is also called receptor-mediated transfer, which increases drug accumulation in tumor tissue and reduces its side effects on normal cells. Furthermore, FA contains functional groups (carboxyl, amine) that can interact with NP surfaces^17^.

Crocin is the most abundant antioxidant compound of saffron with different medicinal effects such as high antioxidant capacity^18^, anti-inflammatory effects^19^, anti-cancer effects by inhibiting the proliferation of cancer cells 13, reducing blood fat^20^, etc. However, crocin’s high sensitivity to degradation by environmental factors and its instability at different pH limits its clinical use. For this reason, in this study, for the first time, modified cellulose crystalline nanocarriers were used for encapsulation and transfer to cancer cells^21^.

## Results

### Characterization of NCC

**Figure 1:**
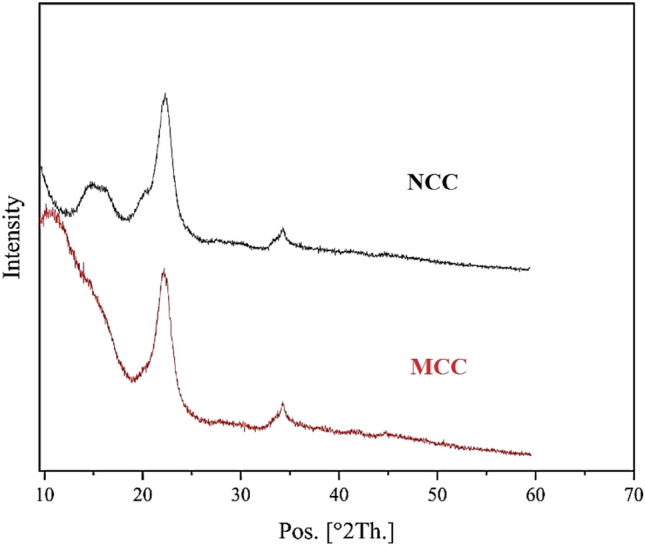
X-ray diffraction (XRD) spectrum for Nano-crystalline cellulose (NCC) and Micro crystallinecellulose (MCC).

Figure 1 revealed the X-ray diffraction (XRD) spectrum for Nano-crystalline cellulose (NCC) and Microcrystalline cellulose (MCC). In the XRD spectra for NCC and MCC, the presence of sharp peaks at 2*θ* = 22.5° indicates the presence of crystalline structures within the cellulose samples. This peak suggests that both NCC and MCC possess high crystallinity and regular arrangement of cellulose molecules within the crystalline regions. Furthermore, the appearance of an additional peak around 15° in the XRD spectrum of NCC indicates the presence of a hydrogen bonding network within the cellulose structure. This peak is often associated with the ordered arrangement of cellulose molecules and specific crystalline planes within the structure. In the case of cellulose, the hydrogen bonding interactions play a crucial role in stabilizing the crystalline structure and contributing to the overall stability of the material. The appearance of this peak in the XRD spectrum suggests that NCC exhibits a well-defined hydrogen bonding network. Additionally, NCC has a significantly smaller crystal size than MCC. Since NCC has a higher surface area-to-volume ratio than MCC, the hydrogen bonding in this plane becomes more prominent, leading to a stronger signal in the XRD spectrum. Besides, the higher Crystallinity Index (CrI) in NCC (66.6%) compared to MCC (61.4%) is a consequence of the acid hydrolysis process in NCC synthesis. This process selectively removes amorphous regions, leading to a more crystalline structure with a higher CrI^8,22^.

### Characterization of NCF-CR-NPs

#### Dimensions of NPs

**Figure 2:**
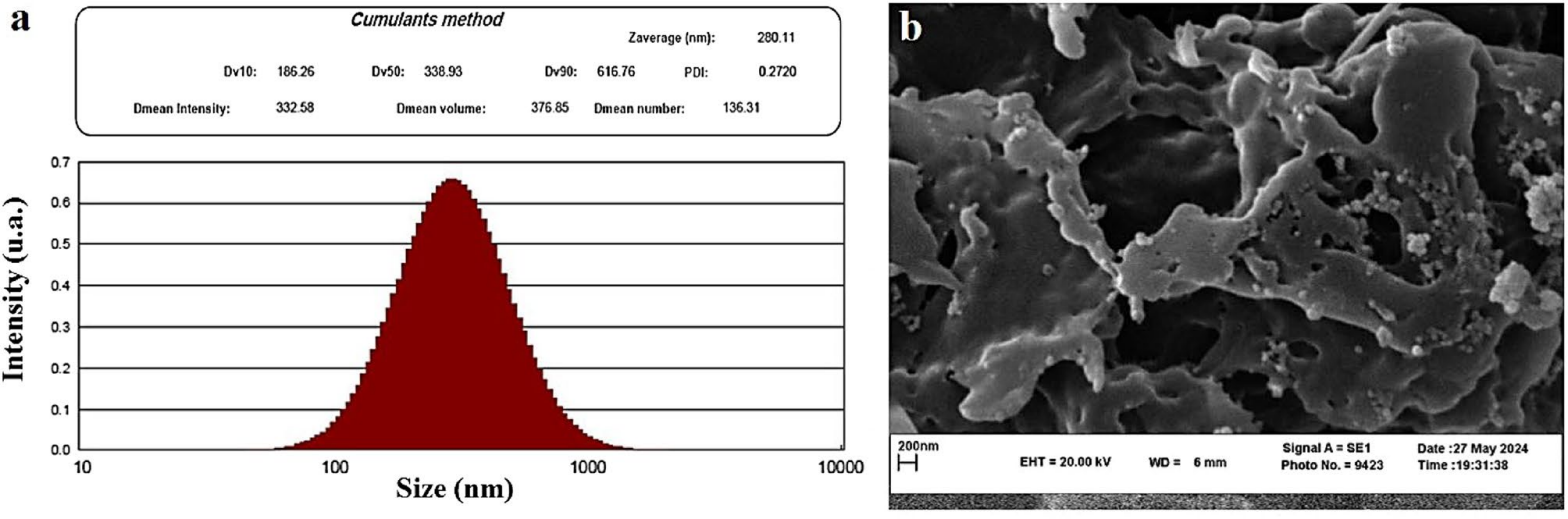
Determining the dimension of NPs. (**a**) DLS assay for an NCF-CR-NP. The Z-average, D mean, and PDI values were 280.11 nm, 136.31, and 0.27, respectively. (**b**) SEM micrograph showing the dispersed and well-separated NCF-CR-NPs with uniform and regular morphology.

The dynamic light scattering (DLS) assay was utilized to determine the size of nanoparticles (Fig. 2a). According to the result, the mean actual diameter (D mean) and mean hydrodynamic size (Z-average) of NCF-CR-NPs were 124.5 ± 7.1 nm and 242.7 ± 24.5 nm, respectively. Also, the mean polydispersity Index (PDI) of the NPs was 0.225 ± 0.03, indicating monodisperse particles. Moreover, the surface charge of the NPs was evaluated using zeta potential measurements. The average zeta potential values at pH 4 were − 26.25 ± 3.43 mV for NCC, − 18.85 ± 9.72 mV for NCC-CTAB, − 21.36 ± 8.26 mV for crocin, and − 23.87 ± 8.92 mV for NCF-CR-NPs. As can be seen, the modification of NCC using CTAB resulted in a more positive surface charge, which is consistent with the results of similar studies^23^. These results indicate that the synthesized NPs had appropriate stability, as NP stability requires a high negative or positive zeta potential. This high zeta potential promotes electrostatic repulsion between particles with similar charges, preventing their aggregation. Therefore, the NCF-CR-NPs were considered to be stable particles with sufficient force to resist agglomeration. In addition, the SEM micrograph (Fig. 2b) showed dispersed and well-separated NCF-CR-NPs, with a uniform and regular morphology, without significant aggregation or agglomeration. These characteristics would align with the DLS results and confirm the stability and monodispersity of the synthesized NCF-CR-NPs.

#### FTIR

**Figure 3:**
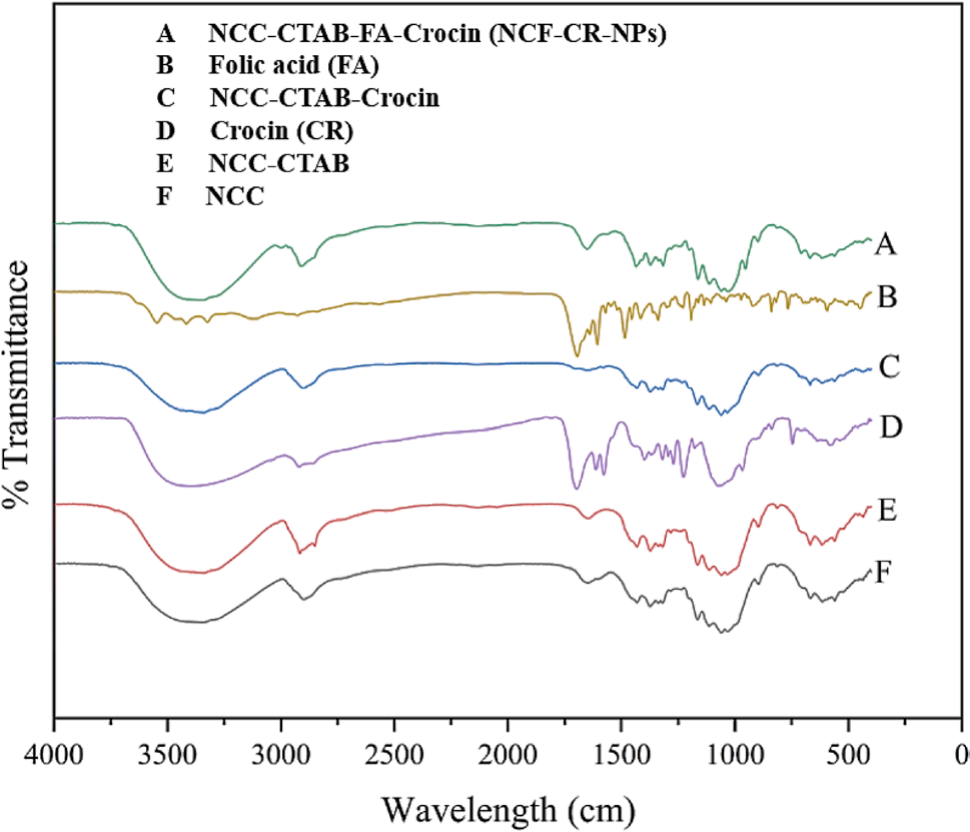
FTIR spectra of NCC, NCC-CTAB, crocin (CR), NCC-CTAB-CR, Folic acid (FA) and NCF-CR-NPs. The diagram shows the transmittance (%) versus wavelength for crystal structures.

Figure 3 shows the FTIR spectrum associated with different compounds used in the synthesis of NPs and the spectrum of the synthesized NP (Fig. 3). The hydrophilic head of CTAB (cationic headgroup) interacts with the surface of the NPs, which may have been functionalized or inherently negatively charged. Also, the long hydrophobic tails of CTAB create a surrounding layer around the NPs, stabilizing them in aqueous solutions. As shown in the diagram, the difference between the spectrum of NCC and NCC-CTAB is related to the formation of a peak at 2850 cm^−1^ (corresponding to CH_2_ vibrations) and the change of the peak band in the area of 1647 cm^−1^, which has been changed to 1648 cm^−1^. Also, the bandwidth variable in 3341 cm^−1^ indicates the binding of NCC with CTAB. The presence peak at 1431.6 cm^−1^ is related to the trimethyl groups of quaternary ammonium in CTAB that finally confirmed the formation of NCC-CTAB^8^.

FA can interact with the NP surface through its carboxylate groups, forming ionic bonds with positively charged surfaces modified by CTAB. The amine groups (–NH_2_) of FA might form hydrogen bonds with surface groups on the NCF-CR-NP. The vibrational spectrum of FA is shown in Fig. 3. The presence of a peak at 1694 cm^−1^ is related to C=O stretching, the peak at 1606 cm^−1^ is related to the bending state of the N–H group, and peaks in the range of 1200 to 800 cm^−1^ corresponded to the absorption of the phenyl ring (aromatic C=C bending) of carbohydrate bands and also related with stretching vibrations of C–O and C–C groups and bending vibration of C–O–H group. The band in the region of 765 cm^−1^ is characteristic of FA and is associated with C–H aromatic bending^24^. From the characteristic bands of pure crocin, we can mention the bands in the region of 1073 cm^−1^ and 1612 cm^−1^. The peak at 1073 cm^−1^ is related to C–O sugar groups of crocin^25^. Examining the peaks in the NP shows the presence of the characteristic peaks of all the used compounds with changes. The fact that the peaks in the NP are shifted compared to the spectra of the pure compounds indicates the interaction between the NP components (covalent and non-covalent bonds)^25^.

#### FESEM results

**Figure 4:**
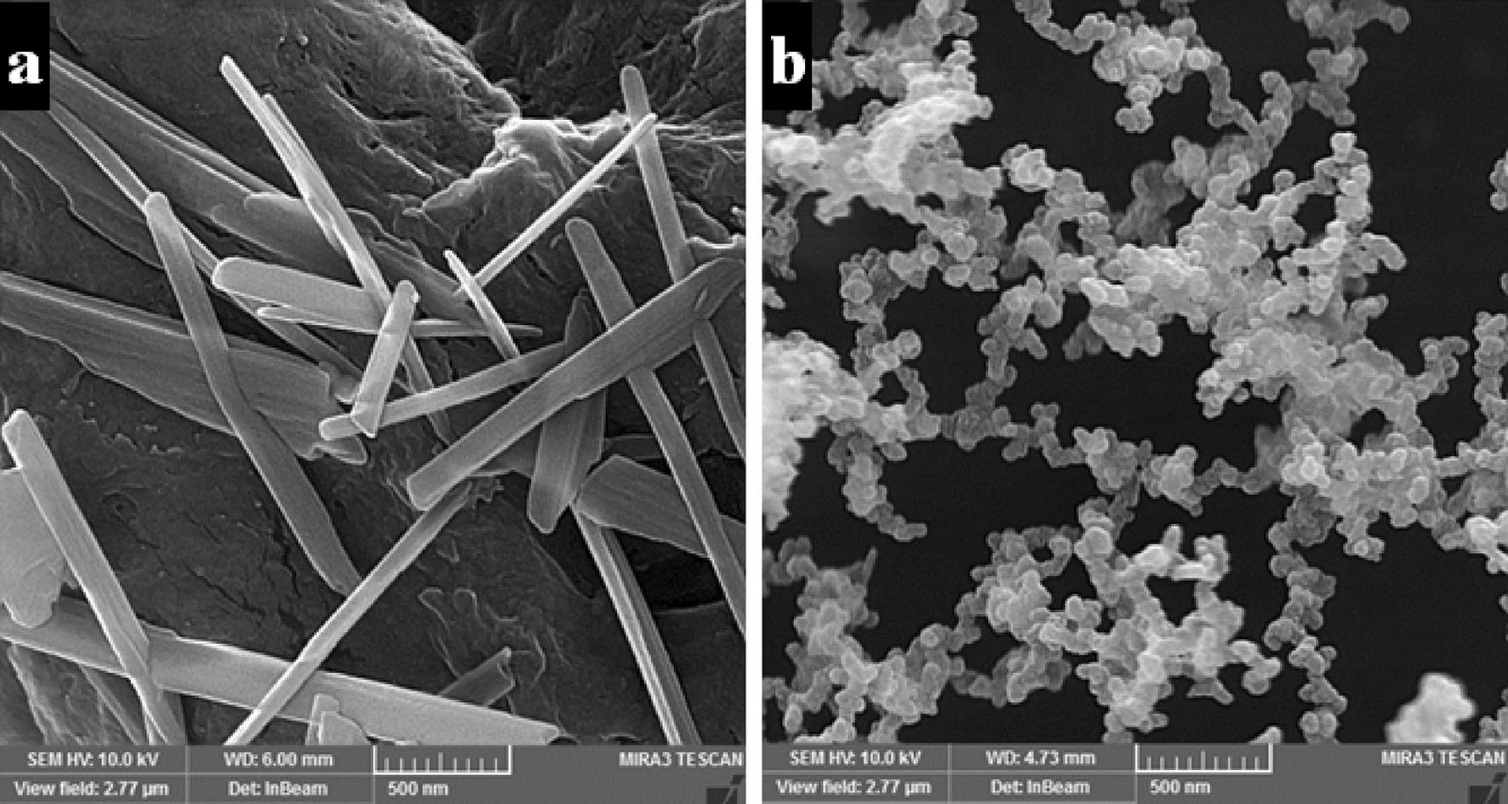
Field Emission scanning electron microscopy (FESEM) micrograph of (**a**) NCC, and (**b**) NCF-CR-NPs.

Analysis of the results of the morphology of NPs was done by the FESEM method. The results of this investigation showed elongated and needle-shaped morphology of cellulose nanocrystals (Fig. 4a). Comparing the images of cellulose nanocrystals with the images of surface-modified nanocrystals containing drugs (Fig. 4b) shows a clear morphological change.

### Encapsulation efficiency

The drug encapsulation efficiency and loading capacity of the NPs were determined using spectrophotometry (Supplementary Fig. S1). A standard curve was generated by measuring the absorbance of various concentrations of crocin at 443 nm. The encapsulation efficiency and drug loading were determined using the standard curve. The encapsulation efficiency of 88.17%, indicates that a large fraction of the crocin was successfully incorporated into the NP structure. Additionally, the loading capacity was determined to be 12.59%, suggesting that the NPs could include the appropriate amount of the crocin. These findings demonstrate the efficient encapsulation of the crocin within the NP system, a crucial prerequisite for effective drug delivery and targeted therapy. These results confirmed that the NP could effectively solubilize and incorporate the hydrophobic crocin, enhancing its therapeutic potential.

### In vitro cytotoxicity assay

**Figure 5:**
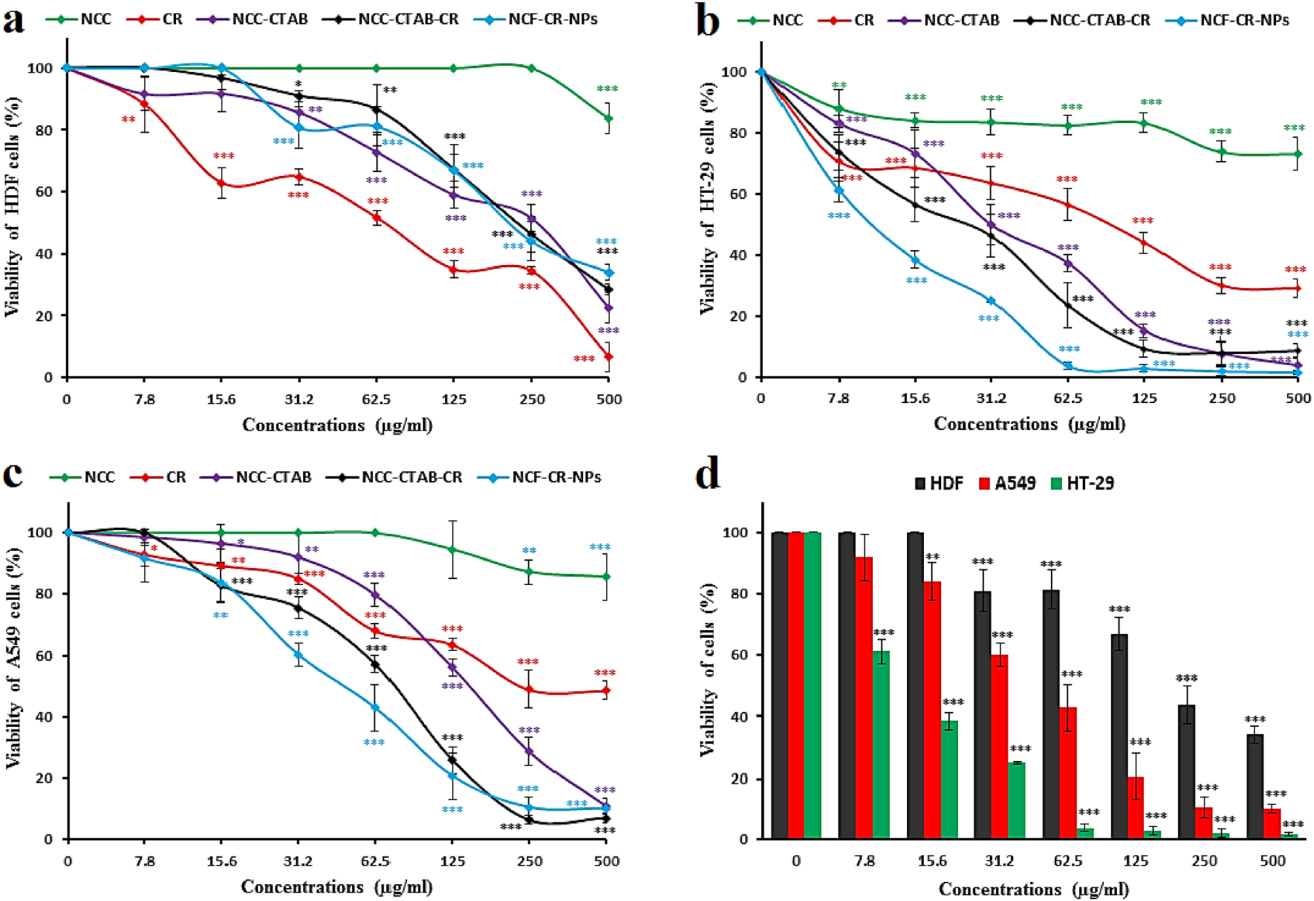
Cytotoxicity assay of NCC, crocin (CR), NCC-CTAB-CR (without FA), and NCF-CR-NPs at concentrations of 0, 7.8, 15.6, 31.2, 62.5, 125, 250, and 500 μg/ml for 48 h by MTT test on the HDF (human dermal fibroblasts) cells (**a**), HT-29 (colon cancer) cells (**b**), and A549 (lung cancer) cells (**c**). The comparison of the cytotoxicity of NCF-CR-NPs on the three cell lines is shown in (**d**). The results demonstrated that NCF-CR-NPs exhibited significantly higher toxicity against the HT-29 colon cancer cells, which have positive FR expression. The data are expressed as mean ± standard deviation (SD), and the statistical significance is indicated as **P < 0.01 and ***P < 0.001 compared to the untreated (control) cells.

As shown in Fig. 5, the cytotoxicity effect of NCC, crocin (CR), NCC-CTAB-CR (NCC containing crocin without FA), and NCF-CR-NPs, using the MTT assay. The toxicity was evaluated on normal cells (HDF—human dermal fibroblasts) (Fig. 5a) and cancer cells with positive (HT-29—human colorectal adenocarcinoma) (Fig. 5b), and negative (A549—human alveolar adenocarcinoma) FR expression (Fig. 5c). The results showed that crocin and NCC exhibited relatively low toxicity towards the cancer cell lines. However, both NP formulations containing crocin (NCC-CTAB-CR and NCF-CR-NPs) demonstrated significant and dose-dependent cytotoxicity on cancer cell lines. In addition, the comparison between the two NP formulations, NCF-CR-NPs (containing FA) and NCC-CTAB-CR (without FA), revealed that the cytotoxicity of NCF-CR-NPs against the FR-positive HT-29 cells was significantly higher than its effects on the FR-negative A549 cells. Additionally, the NCF-CR-NPs had a stronger cytotoxic impact on the HT-29 cells (FR-positive) than the A549 cells (FR-negative). In contrast, the effects of both NP formulations on the A549 cells were similar. These data suggest that the presence of FA in the NP structure (NCF-CR-NPs) enhances the cytotoxic effect on the FR-positive cells, likely due to the increased internalization of the NPs through FR-mediated endocytosis. The dose-dependent cytotoxicity profile observed in Fig. 5d further supported the potent cytotoxicity of NCF-CR-NPs on the HT-29 cells in a concentration-dependent manner. The dose-dependent cytotoxicity of the NCF-CR-NPs against the HT-29 cell line further confirms the potential of this NP for selective cancer cell killing.

#### Evaluation of IC_50_

As shown in Table 1, the assessment of the IC50 for NCC, crocin (CR), NCC-CTAB-CR (NCC containing crocin without FA), and NCF-CR-NPs further corroborated the findings from the MTT assay. The IC50 assay revealed that the encapsulation of crocin within the NP structure (NCF-CR-NPs) diminished the IC50 value compared to the free crocin form. This indicates that the NP formulation enhanced the cytotoxicity of crocin. Furthermore, the IC50 of the NCF-CR-NPs against the FR-positive HT-29 cells was significantly lower than the IC50 of the FR-negative A549 cells. This selective and enhanced cytotoxicity of the NCF-CR-NPs towards the HT-29 cells is consistent with the MTT assay results. These findings suggest that including FA in the NP design (NCF-CR-NPs) facilitated the targeted delivery and enhanced the cytotoxicity on the FR-expressing cancer cells.

**Table 1:** The IC50 of particles against normal and cancer cell lines.

IC50 (μg/ml)	Crocin	NCC	NCC-CTAB	NCC-CTAB-CR	NCF-CR-CPs
HT-29 (FR +)	95.27	< 500	31.13	25.46	11.6
A549 (FR−)	255.18	< 500	153.16	76.94	49.67
HDF (Normal)	68.95	< 500	262.93	228.5	216.94

### Apoptosis assay

#### AO/PI staining

**Figure 6:**
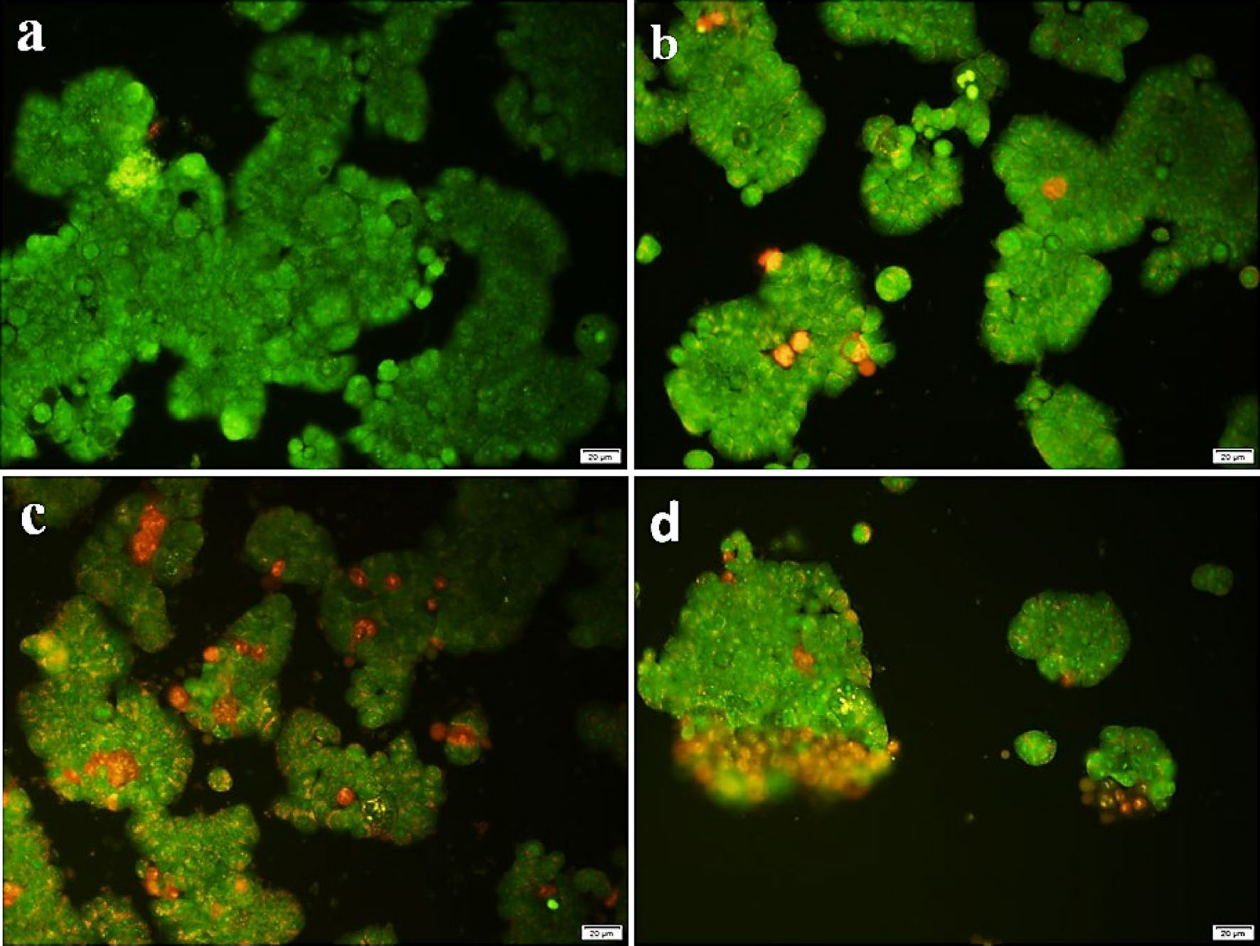
Fluorescence microscopy images of HT-29 (colon cancer) cells stained with acridine orange (AO) and propidium iodide (PI) to assess cell viability after treatment with the NP formulations. AO stains live cells with green fluorescence, while PI stains apoptotic cells with red fluorescence. The cells were exposed to (**a**) no treatment (control), (**b**) 6 μg/mL NCF-CR-NPs, (**c**) 12 μg/mL NCF-CR-NPs, and (**d**) 24 μg/mL NCF-CR-NPs.

The AO/PI staining of HT-29 colon cancer cells showed that the untreated cells (Fig. 6a) exhibited predominantly green fluorescence, indicating a high proportion of viable cells. As the concentration of the NCF-CR-NPs increased, a gradual shift towards more red fluorescence was observed, indicating a higher proportion of apoptotic cells. In the cells treated with 6 μg/mL of NCF-CR-NPs (Fig. 6b), cells exhibited predominantly green fluorescence, but some red-stained apoptotic cells were also visible. At the 12 μg/mL (Fig. 6c) and 24 μg/mL (Fig. 6d) NP concentrations, a significant increase in red-stained apoptotic cells was observed, suggesting a dose-dependent induction of apoptosis. These results indicate a dose-dependent cytotoxic effect of the NCF-CR-NPs on HT-29 colon cancer cells.

#### Flow cytometry and annexin V/PI staining for cell apoptosis analysis

**Figure 7:**
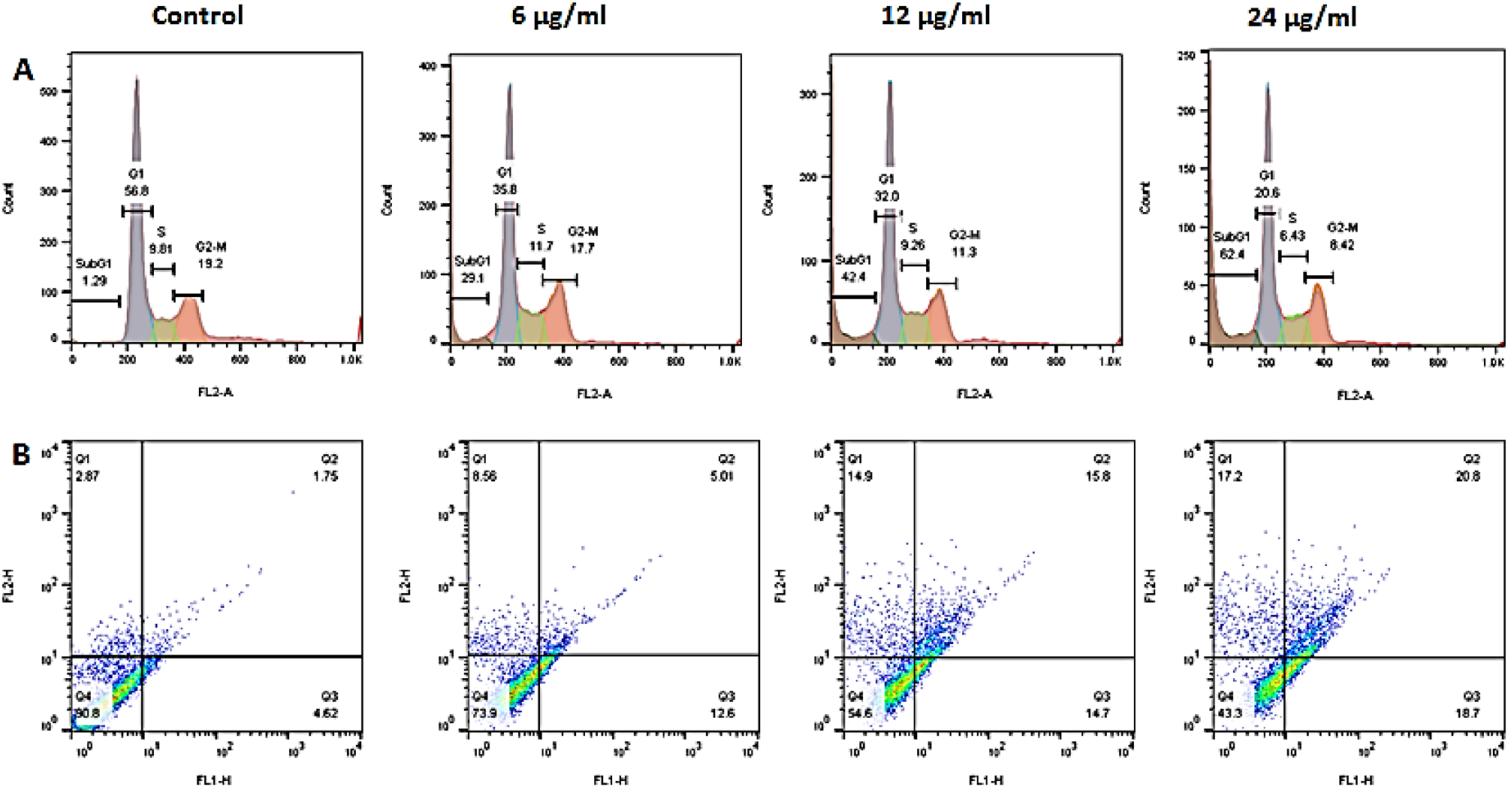
Confirmation of increased cell death rate by (**A**) flow cytometry and (**B**) annexin V- PI staining.

Flow cytometry results (Fig. 7a) showed an increase in the percentage of apoptotic cells. As shown in the images, the amount of subg1 in untreated cells is 1.29%, while in cells treated with concentrations of 6, 12, and 24 μg/ml; the percentage of inhibition is 29.1, 42.2 and 62.4%. Since the SubG1 phase indicates the percentage of apoptotic cells, the results show an increase in the amount of apoptosis during the increase in the treatment concentration. Annexin staining results (Fig. 7b) also showed an increase in the percentage of apoptotic cells during the increase in treatment concentration. According to these results, the highest percentages of apoptotic cells have been reported in the primary and secondary stages. At the highest treatment concentration, 57% of the cells have suffered cell death, of which only 17% have been reported as necrosis.

#### Quantitative analysis of apoptosis genes

**Figure 8:**
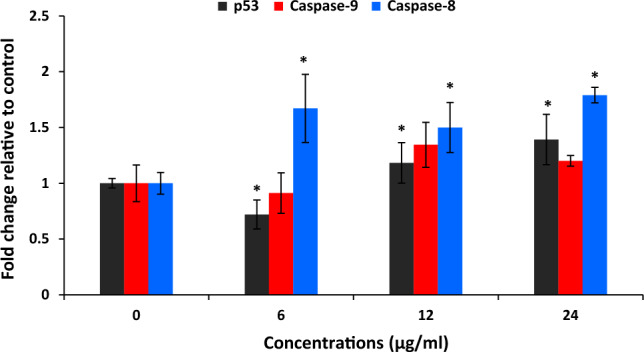
Quantitative evaluation of the expression of apoptosis-related genes in cells treated with the NCF-CR-NPs. The data are presented as mean ± standard deviation (SD), and the statistical significance is denoted by *P < 0.05 compared to the untreated control group.

Examining the change in the expression of apoptotic genes showed the activation of the extrinsic pathway of apoptosis with an increase in the expression of the caspase 8 gene. In cells treated with concentrations of 12 and 24 μg/ml, a significant increase in the expression of the P53 gene was observed (Fig. 8), which indicates the activation of the apoptotic pathway in cells treated with NPs.

### Antioxidant capacity of NCCF-NPs

**Figure 9:**
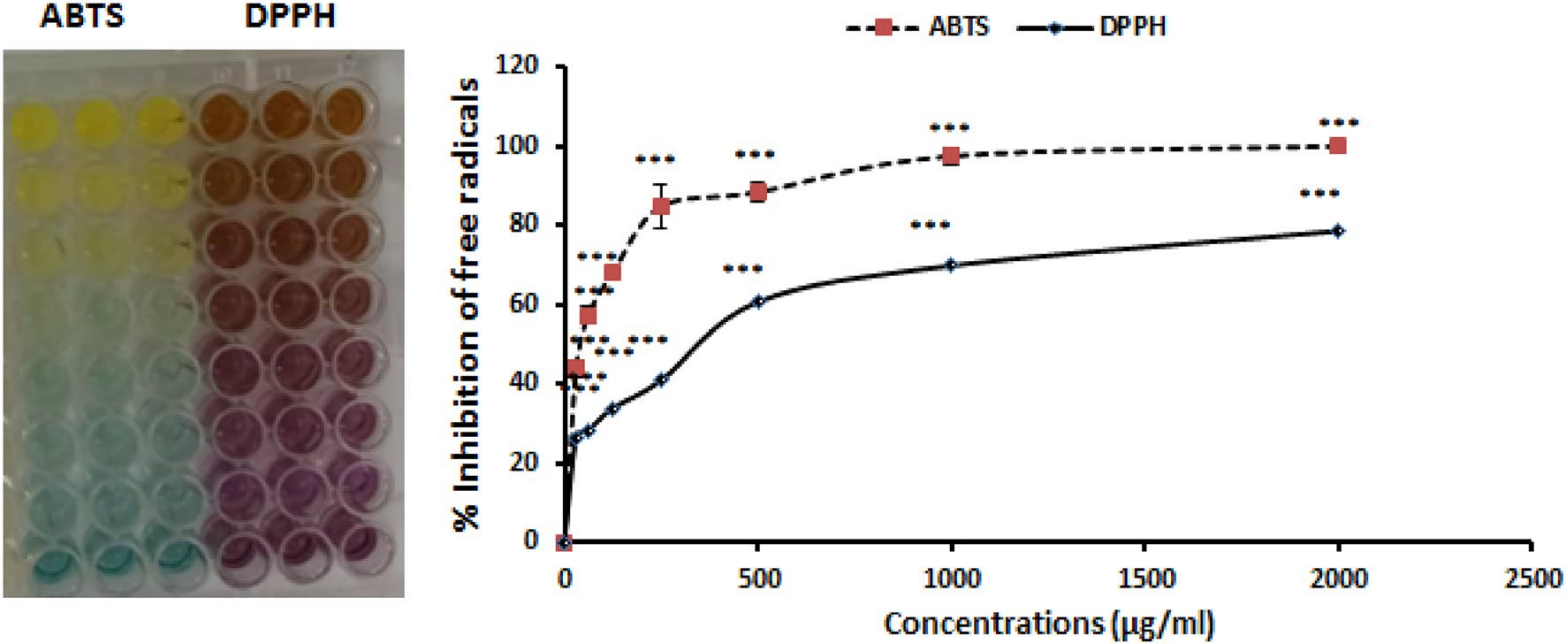
Antioxidant capacity of NPs: increasing the ability to inhibit ABTS and DPPH free radicals by increasing the concentration of NPs. *P < 0.05, **P < 0.01and ***P < 0.001, ± SD.

The inhibition ability of ABTS and DPPH free radicals by NPs was evaluated in laboratory conditions and the results showed a higher inhibitory effect of NPs on ABTS free radicals. According to the results, at a concentration of 46.52 μg/ml of NPs, about 50% of ABTS free radicals were inhibited, while this amount of DPPH radicals was inhibited at a concentration of 369.6 μg/ml of NPs (Fig. 9). These results show the high antioxidant power of NPs on inhibiting free radicals of ABTS.

## Discussion

Colorectal cancer is a common and deadly malignancy, often resistant to current treatment options^26^. While early-stage cases may be treated with conventional clinical treatments, the metastatic form of the disease typically does not respond appropriately to the available interventions^27^. As a result, the development of novel therapies is crucial to Treatment-resistant colorectal cancer. In this regard, the present study aimed to synthesize and characterize a novel nano-system based on NCC, CTAB, and FA for targeted delivery of crocin to cancer cells. FRs are overexpressed on the surface of various cancer cells, including colorectal cancer cells, making folic acid an ideal targeting moiety for drug delivery. The synthesized nano-system, designated NCF-CR-NPs, demonstrated promising features for cancer therapy. According to our findings, NCC possesses high crystallinity and regular arrangement of cellulose molecules within crystalline regions. NCC exhibited a higher crystallinity index (CrI) than MCC, indicating a more crystalline structure. The synthesized NPs had appropriate stability with a monodisperse particle size. The encapsulation efficiency of crocin within the NPs was high, indicating successful incorporation. Results from cytotoxicity assays showed that NP formulations containing crocin (NCF-CR-NPs) exhibited significant dose-dependent cytotoxicity on cancer cells, with enhanced effects on FR-positive cells when folic acid (HT-29 colorectal cancer cells) was included in the NP structure. Compared to free crocin, this enhanced cytotoxicity can be attributed to the targeted delivery mechanism facilitated by incorporating FA. The observed difference in cytotoxicity between HT-29 and A549 cells highlights the targeting capability of the NCF-CR-NPs. A549 cells, lacking FRs, showed significantly lower sensitivity to the nanocarrier, indicating that the observed cytotoxicity is primarily attributed to the targeted delivery mediated by folic acid. This selective targeting ability is crucial for minimizing off-target effects and maximizing the therapeutic index. Additionally, the higher IC50 value for NCF-CR-NPs compared to free crocin further supports the hypothesis that the nanocarrier enhances the cytotoxic effects of crocin, probably by facilitating its internalization within the target cells. Also, the evaluation of apoptotic cells indicated a dose-dependent induction of apoptosis by the NPs in cancer cell lines. Additionally, the NPs showed high antioxidant power in inhibiting free radicals. Overall, these findings suggest the potential of this nanocarrier for targeted drug delivery, minimizing off-target effects, and enhancing therapeutic efficacy.

Crocin and its ester compounds are among the most significant water-soluble carotenoids. Various studies have investigated crocin’s abilities to inhibit free radicals^28,29^, as well as its therapeutic benefits in treating coronary heart disease^30^, diabetes^31,32^, and cancer^33,34^. For example, Aung et al.^35^ investigated the anti-proliferative effects of crocin on three colorectal cancer cell lines (HCT-116, SW-480, and HT-29), as well as non-small cell lung cancer (NSCLC) cells and non-cancer cells. According to their results, crocin at 1.0 mM significantly reduced proliferation in the HCT-116, SW-480, and HT-29 cells to 2.8%, 52%, and 16.8%, respectively (*P* < 0.01). The anti-proliferative effect was also observed in NSCLC cells, but crocin did not significantly affect the growth of non-cancer young adult mouse colon cells. This study’s results regarding crocin’s toxicity on colorectal cancer cell lines are comparable to ours^35^. In another study, Fanayi et al.^36^ examined the effects of crocin, the chemotherapy drug doxorubicin, and radiation on the MCF-7 breast cancer cell line, individually and in combination. The results showed that crocin could decrease MCF-7 cell growth in a dose-dependent manner. Furthermore, crocin in combination with radiation or doxorubicin resulted in a high apoptosis rate compared to the individual treatments, as demonstrated by flow cytometry and western blot analysis of apoptotic proteins. The researchers concluded that the combined therapy of crocin with radiation or doxorubicin was more effective at inducing apoptosis in the MCF-7 breast cancer cells than the individual treatments alone, suggesting the potential for crocin therapies in breast cancer^36^. Similar to our research, this study also revealed a crocin concentration-dependent toxicity (IC50 of 3.5 mg/ml); nevertheless, our study documented significantly greater toxicity of free crocin and crocin loaded in NPs towards colorectal cancer cells with an IC50 of 68.95 µg/ml and 216.94 µg/ml, respectively. Many other studies also report the cytotoxic effect of crocin against colon^37^, prostate^38^, blood^39^, bladder^40^, ovary^41^, gastric ^34^, and lung^42^ cancers. Although crocin possesses several beneficial properties, including antioxidant capabilities, anti-tumor properties, and anti-atherosclerotic effects, its clinical use faces various limitations. Crocin is susceptible to degradation when subjected to light, heat, and acidic pH. Moreover, the instability of crocin in gastric pH, hydrophilic characteristics, and high molecular weight all contribute to its limited bioavailability and inadequate absorption through the intestinal epithelium. The enzymatic degradation of crocin in the gut further reduces its overall biological effectiveness and therapeutic value^21^. Using cellulose nanocarriers is a good solution to overcome these limitations.

The nanoscale drug delivery systems can facilitate tumor targeting through the enhanced permeability and retention (EPR) effect^43^. Additionally, the small size of NPs enables more effective interaction with tumor cells. Previous studies have reported the anticancer properties of crocin, as well as the effects of Nanosystems carrying crocin^21,44^. However, no research has been conducted on cellulose nanocarriers modified with CTAB as a cationic surfactant and FA to deliver crocin to cancer cells. Despite the advantages of NCC as a drug delivery system, their surface hydroxyl groups limited their therapeutic application. Modifying the surface of NCC can be an effective approach to address these limitations. In this regard, Jackson et al. (2011) used the cationic surfactant CTAB to modify the surface of NCC NPs, which resulted in increased drug loading capacity and sustained release of anticancer agents^9^. Other research has also confirmed the significance of this carrier system for the delivery of different medications^8,45^. In the current study, we conjugated the CTAB-modified nanocrystal with FA to facilitate targeted delivery to FR-expressing cancer cells. We synthesized NCC from MCC, according to the methods described in previous studies^22,46^. The XRD results confirmed the presence of the characteristic peaks associated with NCC. Subsequently, the surface of the NCC was modified with the cationic surfactant CTAB. The FTIR results showed the presence of functional groups as previously reported^46^. Finally, the loading of the crocin onto the CTAB-modified NCC was carried out, and FTIR characterization of the NPs confirmed the successful encapsulation of the crocin. Subsequently, the NPs were conjugated with activated FA. Particle size assessment via DLS indicated an increase in the hydrodynamic diameters of NCF-CR-NPs compared to unmodified NCC (Fig. 1a,b). Zeta potential analysis confirmed the negative charge of NCC-CTAB, CR, and NCF-CR-NPs, which remained consistent with the negative charge of unmodified NCCs. Furthermore, electron microscopy revealed a morphological shift of NPs from elongated to spherical state in NCF-CR-NPs (Fig. 4a,b). NCF-CR-NPs showed the highest toxicity of 23% at 500 μg/ml against HT-29 cells. CTAB-modification of the NCC NPs decreased cell viability across all cell lines tested. Free crocin had an IC50 of ~ 95 μg/ml against HT-29 cells, while the NCF-CR-NPs demonstrated the greatest toxicity against HT-29 cells, with an IC50 of ~ 11 μg/ml. This heightened toxicity in the HT-29 cells compared to A549 cells (IC50 ~ 50 μg/ml) confirmed the role of FR in colorectal cancer cells. Fluorescent staining and flow cytometry analysis demonstrated that the NCF-CR-NPs treatment induced apoptosis in a dose-dependent manner. At a concentration of 6 μg/ml, around 26% of cells underwent apoptosis, with 13% in the early stages of apoptosis. At the IC50 concentration, 46% of cells were killed, with equal distribution between early apoptosis, late apoptosis, and necrosis. At higher concentrations, most apoptotic cells were in the secondary apoptosis stage. In addition, treatment of HT-29 cells with NCF-CR-NPs at concentrations of 12 and 24 μg/ml resulted in increased expression of caspase 8 and p53 genes, indicating the activation of the apoptosis pathway. Similarly, in a study published in 2018, Wang and colleagues via flowcytometry analysis revealed that treatment with crocin led to cell cycle suppression in the G0/G1 phase of skin cancer cell lines A431 and SCL-1^47^, which confirms the role of CR in suppressing the growth and proliferation of cancer cells. Several studies also have shown the crocin’s ability to inhibit cell proliferation and induction of apoptosis via increasing the expression of BAX and p53 as well as activating caspase 8 in cancer cells, which is consistent with the present study^34,38,48,49^.

Overall, this research highlights the potential of utilizing NCC-CTAB-based nanocarriers as a platform for targeted drug delivery. The successful incorporation of FA and crocin into this system offers a promising approach for developing an effective targeted therapy. Future studies focusing on i*n vivo* efficacy and biocompatibility will provide further insights into the potential of NCF-CR-NPs for clinical applications.

## Materials and method

### NCC prepared from MCC and characterization

MCC was used to prepare NCC. The treatment was done with 64% by weight sulfuric acid. For this purpose, 10 gr of MCC was added in sulfuric acid and the mixture was incubated for 90 min at 60 °C under stirring. After the incubation, to stop the reaction, 800 ml of cold distilled water was added to the mixture and the hydrolyzed suspension was centrifuged and washed several times. Thereafter, the suspension was dialyzed for 7 days and subsequently ultrasonically dispersed to form a homogeneous white suspension. Finally, the sample was lyophilized^50^.

#### X-ray powder diffraction and Relative crystallinity Analysis of MCC and NCC

XRD method was used to investigate the structure and Relative crystallinity (RC) of MCC and NCC. The scan obtained from the device was recorded in the range of 2*θ* from 10 to 70 degrees. The RC of MCC and NCC was determined according to Ge et al.^51^:

$${\text{RC }}\left( \% \right) \, = {\text{ Ac}}/ \, \left( {{\text{Ac }} + {\text{ Aa}}} \right) \, \times \, 100$$

A_c_ = Crystalline surface area; A_a_ = total area.

#### Characterization of NCC by FTIR, DLS, and FESEM

For imaging, the powder prepared from cellulose nanocrystals was fixed on a grid as a single layer. Following coating, it was checked in the imaging device. To measure the surface charge and average particle size, a uniform dispersion of particles was prepared with an ultrasound probe, and the sample was analyzed at a temperature of 25 °C. FTIR technique was used to evaluate and identify the functional groups. The NP powder was combined with potassium bromide and prepared in the form of compressed tablets. Finally, the tablet was placed in the FTIR device and the spectrum was recorded.

### Modification of NCC with CTAB surfactant, characterization and binding of CR

To NCC surface modification with CTAB, 200 mg of NCC was added to the aqueous solution of 4 µM CTAB, and the resulting mixture was placed on a magnetic stirrer for 3 h at 60 °C. Next, the resulting mixture was centrifuged at 12,000 rpm for 10 min to remove free CTAB. The sample was prepared and analyzed to assess the surface charge (Zeta potential) and identify the functional groups and bonds (FTIR) according to the method described in section “Characterization of NCC by FTIR, DLS, and FESEM”. To load CR on NPs, CR was dissolved in deionized distilled water and then added to the NCC-CTAB solution. Finally, the mixture was centrifuged and lyophilized^45^.

### Preparation of the NCC-CTAB-CR functionalized with the FA and characterization of NCF-CR-NPs

FA was first activated with NHS and EDC. The resulting solution was added to the NCC-CTAB-CR solution and incubated for 24 h on a stirrer at room temperature. Then, the resulting mixture was centrifuged and lyophilized^17^.

### Encapsulation and drug loading assay

Encapsulation efficiency (EE) was evaluated by an indirect method. To achieve this, a standard curve was generated based on the consecutive concentrations of crocin, and the formulation of the standard curve was determined. Subsequently, the supernatant obtained from section “Preparation of the NCC-CTAB-CR Functionalized with the FA and characterization of NCF-CR-NPs” was subjected to absorption measurement at the same wavelength as the standard curve, and the quantity of unbound drug was quantified. The quantity of encapsulated drug was calculated by substituting this value into Formula (1). Formula (2) was employed to determine the quantity of drug loaded.

1 $$\% EE = TD{ - }FD/TD \times 100$$

2 $$\% LC = TD{ - }FD/TNPs \times 100$$

### MTT assay

Two cancer cell lines including HT-29 (FR+) and A549 (FR−), as well as, a normal HDF cell line were subjected to MTT assay. The cells were cultured in three replicates in the wells of the 96-well plate and after 24 h, their culture medium was replaced with the treatment medium. 48 h later, the treatment medium was drained and replaced with a specific volume of MTT. After the time needed to perform the MTT dye reaction with the mitochondrial enzymes of living cells, the MTT solution of each well was drained with high precision. Then, 100 μl of DMSO was added to the wells, and finally, the plate was analyzed in an ELISA reader with a wavelength of 570 nm.

### Cell death assay of NCF-CR-NPs

To investigate the type of cell death, flow cytometry was used for cell cycle analysis, Annexin V/PI staining was used to determine the percentage of cells in different phases of cell death, and fluorescence staining was used. Evaluation of changes in the expression of apoptotic genes was also done with a quantitative method.

#### Annexin V/PI

For annexin V/PI staining, cells were cultured in 6-well plates for 24 h and treated for 48 h. Next, the supernatant was drained and the trypsinized cells were transferred to separate microtubes, and centrifuged. The supernatant was drained and 500 μl of cold PBS was added to the sediment of the cells, and after pipetting, the samples were centrifuged. After draining the supernatant, 100 μl of 1X buffer was added to it and vortexed. Next, 1 μl of annexin solution was added to the cell suspension and gently vortexes. After incubating the sample at 25 °C for 15 min in the dark, 1 μl of PI was added, and after overtaxing, the sample was incubated in the dark for 2 min. After incubation, the sample was centrifuged and 400 μl of 1X buffer was added to it, and the samples were analyzed by flow cytometry.

#### Flow cytometry

To perform flow cytometry, after culturing the cells in the 6-well plate for 24 h, the culture medium was replaced with the treatment medium, and the cells were incubated under treatment with different concentrations of NPs for 48 h. After that, the supernatant was drained and the cells were separated from the bottom of the plate with the help of trypsin, and transferred to separate microtubes and then centrifuged at 2800 rpm. The supernatant was drained and the cells were washed twice with PBS. Finally, 300 μl of PI dye was added to each microtube and then the cell suspension containing PI dye was analyzed in a flow cytometry device.

#### Florescent assay

For fluorescent staining, cells were cultured in separate wells and treated with different concentrations of NPs for 48 h. Then, the culture medium of the cells was drained and one milliliter of fluorescent dye was added to each well, and the cells were immediately imaged with a fluorescent microscope. AO/PI dye were used to stain the cells. To prepare 6 ml of AO/PI dye, 6 µl of AO dye (5 mg/ml) and 6 µl of PI dye (3 mg/ml) were added to 6 ml of PBS, and the final dye solution was prepared.

#### Real-time PCR

Quantitative examination of the expression of apoptosis genes was done with the real method. For this purpose, the cells were cultured in a 12.5 flask and after 24 h, different concentrations of NPs were added to each flask in a volume of 4 ml and the samples were incubated for 48 h. Then, the total RNA of the cells was extracted using a special kit and the nanodrop method was used to evaluate the purity and amount of RNA. Then complementary DNA was synthesized using RNA as a template and by the Pars Tous kit. Finally, to evaluate the expression of genes, the synthesized cDNA was used along with special primers, Sybergreen, and water, and the reaction mixture with a volume of 20 μl was analyzed in a BioRAD device.

### Antioxidant assay of NCF-CR-NPs

The antioxidant power of NPs was evaluated by the free radical inhibition method. For this purpose, a specific volume of ABTS and DPPH free radicals was prepared in laboratory conditions, and then different concentrations of NPs with a volume of 100 µl were serially prepared in a 96-well plate in three replicates. Next, 100 µl of free radical color solution were added to each well containing NP solution. NPs exposed to DPPH free radicals were incubated for 30 min in an incubator at 37 °C, and NPs exposed to ABTS free radicals were incubated for 1 h at room temperature in the dark. Finally, the decrease in absorption of DPPH and ABTS free radicals at wavelengths of 517 nm and 730 nm (respectively) was analyzed with a spectrophotometer.

### Supplementary Information

Supplementary Figure S1.

## Data Availability

Additional datasets utilized in this study can be obtained from the corresponding authors upon request.

## References

[1] Park W, Heo Y-J, Han DK (2018). New opportunities for nanoparticles in cancer immunotherapy. Biomater. Res..

[2] Wang R, Billone PS, Mullett WM (2013). Nanomedicine in action: an overview of cancer nanomedicine on the market and in clinical trials. J. Nanomater..

[3] Adair JH, Parette MP, Altınoglu EI, Kester M (2010). Nanoparticulate alternatives for drug delivery. ACS Nano.

[4] Wang X, Zhang H, Chen X (2019). Drug resistance and combating drug resistance in cancer. Cancer Drug Resist..

[5] Dogan M (2022). Assessment of mechanism involved in the apoptotic and anti-cancer activity of Quercetin and Quercetin-loaded chitosan nanoparticles. Med. Oncol..

[6] Robey RW (2018). Revisiting the role of efflux pumps in multidrug-resistant cancer. Nat. Rev. Cancer.

[7] Singh A, Benjakul S, Prodpran T (2019). Ultrasound-assisted extraction of chitosan from squid pen: Molecular characterization and fat binding capacity. J. Food Sci..

[8] Gupta RD, Raghav N (2020). Nano-crystalline cellulose: Preparation, modification and usage as sustained release drug delivery excipient for some non-steroidal anti-inflammatory drugs. Int. J. Biol. Macromol..

[9] Jackson JK (2011). The use of nanocrystalline cellulose for the binding and controlled release of drugs. Int. J. Nanomed..

[10] Khan MR (2024). A review study on derivation of nanocellulose to its functional properties and applications in drug delivery system, food packaging, and biosensing devices. Polym. Bull..

[11] Hu Z, Ballinger S, Pelton R, Cranston ED (2015). Surfactant-enhanced cellulose nanocrystal Pickering emulsions. J. Colloid Interface Sci..

[12] Dutt KR (2017). Method development and validation of etodolac by visible spectroscopy. Indo Am. J. Pharm..

[13] Roman M (2015). Toxicity of cellulose nanocrystals: A review. Ind. Biotechnol..

[14] Bai M, Bornhop DJ (2012). Recent advances in receptor-targeted fluorescent probes for in vivo cancer imaging. Curr. Med. Chem..

[15] Dainty LA (2007). Overexpression of folate binding protein and mesothelin are associated with uterine serous carcinoma. Gynecol. Oncol..

[16] Hartmann LC (2007). Folate receptor overexpression is associated with poor outcome in breast cancer. Int. J. Cancer.

[17] Bittleman KR, Dong S, Roman M, Lee YW (2018). Folic acid-conjugated cellulose nanocrystals show high folate-receptor binding affinity and uptake by KB and breast cancer cells. ACS Omega.

[18] Chen Y (2008). Antioxidant potential of crocins and ethanol extracts of Gardenia jasminoides ELLIS and *Crocus sativus* L.: A relationship investigation between antioxidant activity and crocin contents. Food Chem..

[19] Nam KN (2010). Anti-inflammatory effects of crocin and crocetin in rat brain microglial cells. Eur. J. Pharmacol..

[20] Magesh V, Singh JPV, Selvendiran K, Ekambaram G, Sakthisekaran D (2006). Antitumour activity of crocetin in accordance to tumor incidence, antioxidant status, drug metabolizing enzymes and histopathological studies. Mol. Cell. Biochem..

[21] Tavasoli S (2023). Crocin-loaded nanocarriers; approaches and applications. Curr. Opin. Food Sci..

[22] Fitriani F (2021). Isolation and characterization of nanocrystalline cellulose isolated from pineapple crown leaf fiber agricultural wastes using acid hydrolysis. Polymers.

[23] Putro JN (2019). The effect of surfactants modification on nanocrystalline cellulose for paclitaxel loading and release study. J. Mol. Liq..

[24] Okamoto-Schalch NO (2020). Production and characterization of chitosan-TPP/cellulose nanocrystal system for encapsulation: a case study using folic acid as active compound. Cellulose.

[25] Cordeiro S (2021). Antioxidant-loaded mucoadhesive nanoparticles for eye drug delivery: A new strategy to reduce oxidative stress. Processes.

[26] Samadder NJ (2014). Epidemiology and familial risk of synchronous and metachronous colorectal cancer: A population-based study in Utah. Clin. Gastroenterol. Hepatol..

[27] Hosseini K, Jasori S, Delazar A, Asgharian P, Tarhriz V (2021). Phytochemical analysis and anticancer activity of *Falcaria vulgaris* Bernh growing in Moghan plain, northwest of Iran. BMC Complement. Med. Ther..

[28] Ghadrdoost B (2011). Protective effects of saffron extract and its active constituent crocin against oxidative stress and spatial learning and memory deficits induced by chronic stress in rats. Eur. J. Pharmacol..

[29] Chen, Y. *et al.* Comparative evaluation of the antioxidant capacity of crocetin and crocin in vivo. *Chin. Pharmacol. Bull.* 248–251 (2010).

[30] Zhang R (2009). Comparison of the effects of crocetin and crocin on myocardial injury in rats. Chin. J. Nat. Med..

[31] Costantino L (1999). 1-Benzopyran-4-one antioxidants as aldose reductase inhibitors. J. Med. Chem..

[32] Sepahi S, Golfakhrabadi M, Bonakdaran S, Lotfi H, Mohajeri SA (2022). Effect of crocin on diabetic patients: A placebo-controlled, triple-blinded clinical trial. Clin. Nutr. ESPEN.

[33] Festuccia C (2014). Antitumor effects of saffron-derived carotenoids in prostate cancer cell models. BioMed Res. Int..

[34] Hoshyar R, Bathaie SZ, Sadeghizadeh M (2013). Crocin triggers the apoptosis through increasing the Bax/Bcl-2 ratio and caspase activation in human gastric adenocarcinoma, AGS, cells. DNA Cell Biol..

[35] Aung H (2007). Crocin from Crocus sativus possesses significant anti-proliferation effects on human colorectal cancer cells. Exp. Oncol..

[36] Fanayi AR, Changizi V, Safa M (2016). Effect of crocin and doxorubicin/radiation on the breast cancer cell line, Michigan Cancer Foundation-7. Biosci. Biotechnol. Res. Commun..

[37] Amin A, Bajbouj K, Koch A, Gandesiri M, Schneider-Stock R (2015). Defective autophagosome formation in p53-null colorectal cancer reinforces crocin-induced apoptosis. Int. J. Mol. Sci..

[38] D’Alessandro AM (2013). Crocus sativus stigma extract and its major constituent crocin possess significant antiproliferative properties against human prostate cancer. Nutr. Cancer.

[39] Sun Y (2013). Crocin exhibits antitumor effects on human leukemia HL-60 cells in vitro and in vivo. Evid. Based Complement. Altern. Med..

[40] Zhang Z, Wang C-Z, Wen X-D, Shoyama Y, Yuan C-S (2013). Role of saffron and its constituents on cancer chemoprevention. Pharm. Biol..

[41] Mahdizadeh S (2016). Crocin suppresses multidrug resistance in MRP overexpressing ovarian cancer cell line. DARU J. Pharm. Sci..

[42] Rezaee R (2014). Crocin effects on human myeloma cells regarding intracellular redox state, DNA fragmentation, and apoptosis or necrosis profile. Jundishapur J. Nat. Pharm. Products.

[43] Ersoz M, Erdemir A, Derman S, Arasoglu T, Mansuroglu B (2020). Quercetin-loaded nanoparticles enhance cytotoxicity and antioxidant activity on C6 glioma cells. Pharm. Dev. Technol..

[44] Naderi R, Pardakhty A, Abbasi MF, Ranjbar M, Iranpour M (2021). Preparation and evaluation of crocin loaded in nanoniosomes and their effects on ischemia–reperfusion injuries in rat kidney. Sci. Rep..

[45] Zainuddin N, Ahmad I, Zulfakar MH, Kargarzadeh H, Ramli S (2021). Cetyltrimethylammonium bromide-nanocrystalline cellulose (CTAB-NCC) based microemulsions for enhancement of topical delivery of curcumin. Carbohydr. Polym..

[46] Xiang S (2019). Cellulose nanocrystal surface cationization: A new fungicide with high activity against *Phycomycetes capsici*. Molecules.

[47] Wang G, Zhang B, Wang Y, Han S, Wang C (2018). Crocin promotes apoptosis of human skin cancer cells by inhibiting the JAK/STAT pathway. Exp. Ther. Med..

[48] Liu D-D (2014). Distinct pro-apoptotic properties of Zhejiang saffron against human lung cancer via a caspase-8-9-3 cascade. Asian Pac. J. Cancer Prev..

[49] Zhao P (2008). Proliferation apoptotic influence of crocin on human bladder cancer T24 cell line. Zhongguo Zhong yao za zhi= Zhongguo zhongyao zazhi= China J. Chin. Mater. Med..

[50] Hachaichi A (2021). Nanocrystalline cellulose from microcrystalline cellulose of date palm fibers as a promising candidate for bio-nanocomposites: Isolation and characterization. Materials.

[51] Ge X (2022). Dielectric barrier discharge cold plasma combined with cross-linking: An innovative way to modify the multi-scale structure and physicochemical properties of corn starch. Int. J. Biol. Macromol..

