# Supplementary material for: Folic acid-modified nanocrystalline cellulose for enhanced delivery and anti-cancer effects of crocin
Source: Sci Rep. 2024 Jun 17;14:13985. doi: 10.1038/s41598-024-64758-2 (PMC11183259; doi:10.1038/s41598-024-64758-2)
Supplement: Supplementary file 1 — Supplementary Figure S1. [file 41598_2024_64758_MOESM1_ESM.docx]

**Supplementary Information**

Supplementary Fig. S1. Standard curve of various concentrations of crocin at 443 nm
